# An optimized fractional order virtual synchronous generator with superconducting magnetic energy storage unit for microgrid frequency regulation enhancement

**DOI:** 10.1038/s41598-025-90483-5

**Published:** 2025-02-20

**Authors:** V. Rajaguru, K. Iyswarya Annapoorani

**Affiliations:** 1 School of Electrical Engineering, Vellore Institute of Technology, Chennai, India; 2 Centre for e-Automation Technologies and School of Electrical Engineering, Vellore Institute of Technology, Chennai, India

**Keywords:** African vulture optimization algorithm, Fractional order virtual synchronous generator, Frequency regulation, Microgrid, Superconducting magnetic energy storage unit, Energy science and technology, Engineering

## Abstract

The Virtual Synchronous Generator (VSG) provides inertia and damping virtually, which plays a crucial role in enhancing the frequency stability of the microgrid. However, the virtual inertia added in the VSG increases the order of the system, which increases the likelihood of output real power fluctuations in the transient state, which is potentially dangerous for system stability. Hence, this paper introduces a new approach for frequency regulation in an isolated microgrid using a Fractional Order Virtual Synchronous Generator (FOVSG) which involves more degrees of freedom, integrated with a Superconducting Magnetic Energy Storage (SMES) unit. The parameters of the FOVSG and Fractional Order Proportional Integral Derivative (FOPID) controllers are optimized by the African Vulture Optimization Algorithm (AVOA) using the Integral Time Absolute Error criterion. The suggested system is simulated using MATLAB for multiple scenarios, and the outcomes are compared with the frequency regulation model using the traditional VSG with SMES unit. The performance of the suggested controller is confirmed further by utilizing a classical proportional integral derivative controller. From the results, the proposed frequency regulation model using an FOVSG and SMES unit combination with FOPID controller has superior system dynamics than the system using a traditional VSG with SMES unit. Furthermore, the suggested system using a FOVSG with SMES unit enhances the frequency responsiveness by 83.61% and 73.38% in maximum overshoot and settling period, respectively.

**Supplementary Information:**

The online version contains supplementary material available at 10.1038/s41598-025-90483-5.

## Background and challenges

The ever-increasing global population and technological advancements have resulted in a huge rise in electricity demand. Microgrids are becoming a viable option for addressing the rising demand for electricity owing to their numerous advantages, such as less pollution, better quality of power, increased versatility, a reliable power source, and a decrease in transmission losses. A microgrid is a small network that primarily consists of multiple micro-sources, energy storage devices, and loads. The microgrid system can function in islanded or grid-connected modes. Frequency regulation of microgrids in isolated mode is normally handled by storage systems and diesel generators. While in grid-connected mode, the main grid takes care of frequencies. As a result, load frequency control (LFC) in an isolated microgrid has more difficulties than in grid-connected mode. The increased integration of RES in microgrids causes disparity between generation and power demand due to the low system inertia and intermittent nature of RES. Because of this disparity, the microgrid experiences severe frequency deviation, which degrades the microgrid’s stability. The VSG emulates the properties of a physical synchronous generator through the implementation of a virtual control loop and a virtual rotor. Regulating large power fluctuations is difficult with conventional VSG control due to the use of constant inertia and damping co-efficient. Moreover, virtual inertia added to VSG results in augmented system order, which causes output power fluctuation and thus affects the stability of the system. The above limitations of VSG can be mitigated with the assistance of FOVSG. The FOVSG with SMES unit helps to augment the frequency stability of the microgrid.

### Literature survey

A list of recent studies on this topic is as follows: A new FOVSG scheme is introduced, and it outperforms the traditional VSG in terms of both stability and performance^1^. A new hybrid optimization technique (a combination of AVOA and Pattern Search (PS) optimization algorithms) is suggested for tuning the FOPID controller settings in a two-area power network (multi-unit) considering Electric Vehicles (EVs)^2^. The traditional VSG with SMES unit is utilized to augment the microgrid’s frequency steadiness. Furthermore, AVOA exhibits superior performance compared to other algorithms investigated, such as Harris Hawks Optimization (HHO), Genetic Algorithm (GA), Grey Wolf Optimization (GWO), and Particle Swarm Optimization (PSO)^3^. The construction of a two-stage controller in a modern power system with VSG is introduced for frequency regulation even at elevated RES levels and EVs^4^. A Cohort Intelligence (CI) optimization based LFC of a single and two-area microgrid using the FOPID controller is investigated, and it outperforms the Proportional Integral Derivative (PID) controller optimized with renowned algorithms like PSO and GA^5^. The load frequency regulation in a power network (hybrid) is improved through optimization of the FOPID using a fragmented swarm optimization technique^6^. The chaos particle swarm optimization is used to optimize the FOPID controller in pumped storage power networks in order to maintain the regional frequency^7^. In contrast to the conventional VSG system, the incorporation of virtual inertia (fractional) via FOVSG has the potential to decrease the system hierarchy which dampens real power fluctuations^8^. A fractional order proportional tilt integral derivative plus one controller is proposed in the automatic generation control design to maintain frequency stability^9^. For the efficient use of tidal turbines in a stand-alone microgrid system, an effective cascaded form of fractional order fuzzy PID integral double derivative controller is recommended^10^.

The AVOA-based cascaded controller is presented as a reliable and efficient solution to the LFC issues in interconnected power systems^11^. An optimal VSG with a hybrid energy storage system is designed in order to improve the frequency responsiveness of the microgrid over a range of disturbances^12^. The implementation of SMES systems for renewable energy applications, together with the associated difficulties and potential avenues for further study, is succinctly and clearly reviewed. Furthermore, in recent years, SMES have become more and more popular for integrating renewable energy. In renewable energy systems, SMES is a viable and cost-effective solution for lowering output power fluctuation, managing frequency, enhancing transient stability, and enhancing power quality^13^. A new cascaded controller of the fractional order type is intended to suppress frequency fluctuations in multi-microgrids^14^. The VSG parameters are tuned with the help of quantitative feedback theory to ensure the system’s stability and performance even under significant reduction in microgrid inertia^15^. The utilisation of a chaotic sine cosine procedure based PID controller for LFC study in an autonomous microgrid is presented^16^. A new metaheuristic algorithm called AVOA is introduced which has low computing complexity and increased adaptability to solve the global optimization problems^17^. The VSG is used in a wind-hydro hybrid power system to mitigate frequency fluctuations and enhance frequency stability^18^. The frequency regulation of the standalone microgrid is addressed by the introduction of new fractional order type cascaded controller based on adaptive fuzzy approach^19^. The power grid with ultra-low inertia uses both the VSG and SMES units to make the frequency more stable even under high-RES penetration and nonlinearities^20^.

An atom search optimization tuned FOPID controller for frequency control in a hybrid power system is proposed to examine the frequency stability via Matignon’s theorem^21^. A salp swarm optimization algorithm tuned cascaded controller for the LFC of an independent microgrid with an EV is developed and studied^22^. The utilisation of four intelligent strategies is implemented in order to optimize the tuning process of the fractional order controller, which is responsible for regulating the microgrid’s frequency fluctuations^23^. An independent multi-microgrid system utilizes the grey wolf optimization technique to optimize the PID controller settings for effective frequency regulation^24^. The VSG is coupled with digital frequency fortification to augment frequency consistency and assure renewable power network reliability, even under the significant proportion of energy derived from renewable sources^25^. The numerous coordination plans of distributed energy resources are employed in an independent hybrid microgrid to address the challenges associated with LFC^26^. The integration of a SMES unit with LFC has been implemented to strengthen the frequency steadiness of the Egyptian power network in response to the increased penetration of RES^27^. A novel two-phase PI controller based on adaptive fuzzy logic is designed to mitigate the impact of disruptions and parameter uncertainties in microgrid frequency control^28^. The integration of distributed generation and EV, along with hybrid energy storage units (ultra-capacitor and SMES) is implemented to enhance the efficiency of automatic generation control in a multiple-source thermal-gas system^29^. The control approaches of H_∞_ and µ-synthesis are implemented to optimize the efficiency of secondary frequency regulation in microgrids. Furthermore, it has been demonstrated that the suggested controller exhibits greater robustness in the presence of numerous disturbances^30^.

A PID controller based on fuzzy logic is utilized in interconnected power networks to manage the frequency fluctuations and the controller’s settings are optimized using a hybrid differential evolution PSO strategy^31^. Single-area LFC for reheat, non-reheat, and hydro turbines are all addressed by a FOPID controller, which exhibits superior robustness in the face of ± 50% parametric uncertainty and possesses enhanced capability for rejecting disturbances compared to existing approaches^32^. A new online intelligent approach that integrates fuzzy logic and PSO approaches is used to optimize the tuning of a widely used PI controller in a microgrid’s LFC^33^. In order to assess the small signal stability, time-domain replication is used in the autonomous hybrid renewable energy network^34^. A new AVOA-based load frequency controller is presented to reduce frequency variability in hybrid thermal power networks with dispersed generation units, and the effectiveness of the suggested controller is confirmed by considering various disruptions^35^. A cooperative control system that uses a robust fractional order strategy to effectively regulate the microgrid’s frequency in the presence of uncertainties. Moreover, the suggested control strategy outperforms both the PID and FOPID controllers^36^. The AVOA-tuned cascaded controller is presented to augment the dynamic performance of the interconnected thermal power networks^37^.

## Research gap and motivation

From the above-detailed literature survey, it is found that a lot of research works are focused only on traditional VSG to mitigate the effect of stability issues caused by the low inertia problem of RES. However, there is a huge potential to work on FOVSG with additional degrees of freedom since very few literatures is present in the field of FOVSG and it provides better inertia response which helps to enhance the system stability^1^. These findings from the literature inspired us to work on the FOVSG with additional degrees of freedom to enhance the frequency regulation in an isolated microgrid by considering a SMES unit.

### Contribution and paper organization

This study’s noteworthy contributions are delineated below:

- An optimized FOVSG technique (considering more degrees of freedom) is employed in conjunction with a SMES unit to regulate the microgrid frequency.
- To the best of the author’s knowledge, no existing literature has been found that specifically addresses the frequency regulation of a micro-grid incorporating a FOVSG-based SMES unit utilizing AVOA.
- The suggested FOPID controller and FOVSG parameters are optimized using the recently created AVOA metaheuristic method.
- Furthermore, the proposed system with FOVSG is simulated against traditional VSG with FOPID and PID controllers to demonstrate its efficacy under different scenarios.

This paper is structured as follows: Segment 2 describes the proposed microgrid frequency regulation model. Segment 3 addresses the modelling of FOVSG with SMES unit. Segment 4 describes the recent algorithm named AVOA. Segment 5 presents the proposed FOPID controller structure and objective function. Segment 6 demonstrates the simulation outcomes and discussions on the results. Finally, the conclusions obtained from the proposed work are summarized in Segment 7.

## Microgrid frequency regulation model

**Fig. 1:**
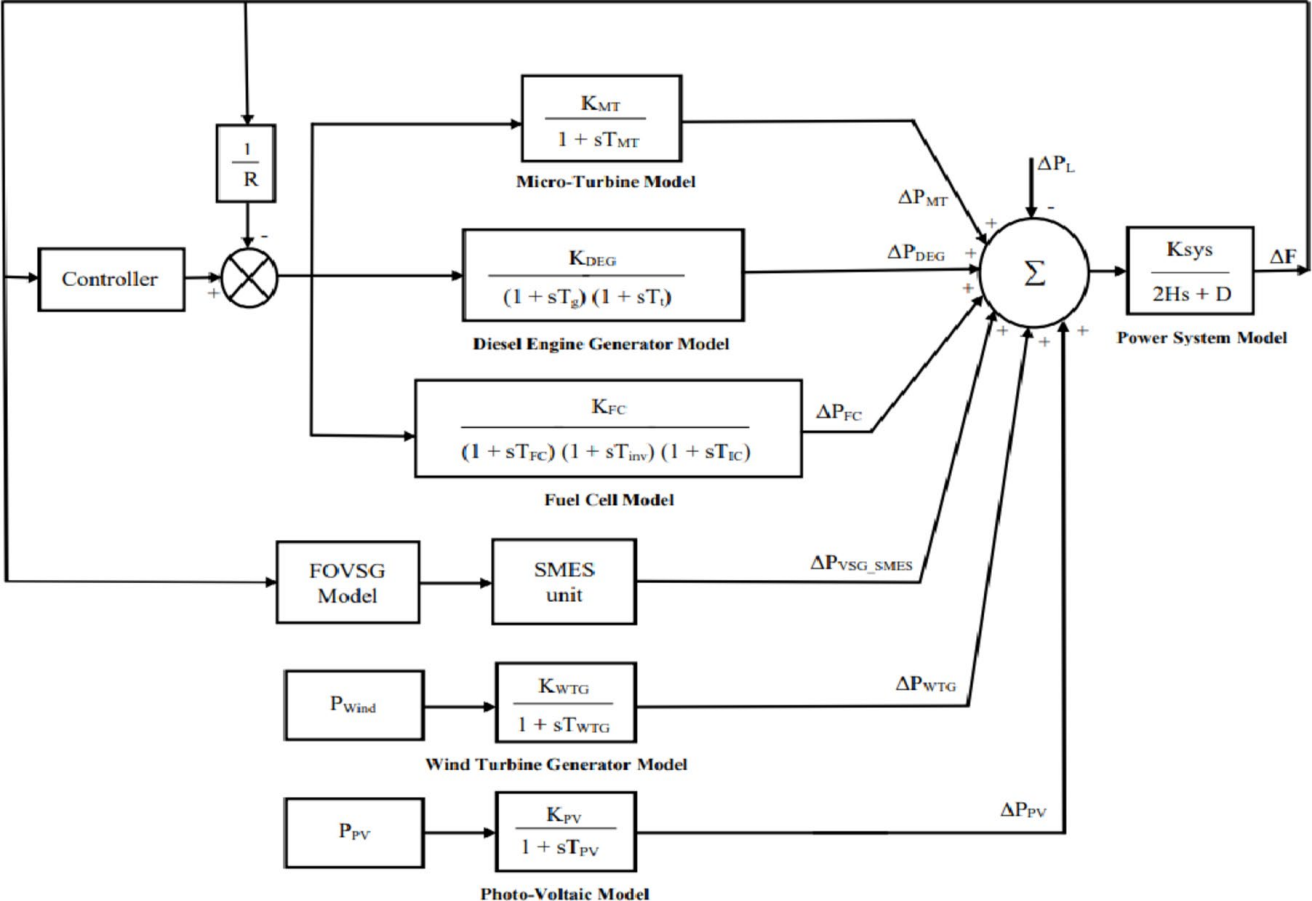
Microgrid frequency regulation model in isolated mode.

The mathematical model of frequency regulation in an isolated microgrid is illustrated in Fig. 1, which comprises a Micro Turbine (MT), Fuel Cell (FC), Diesel Engine Generator (DEG), Wind Turbine Generator (WTG), Photovoltaic (PV) system, FOVSG with SMES unit, and load. The power output of RES (PV and WTG) is unpredictable owing to environmental factors. As a result, they are not included in the LFC loop. The LFC controller controls the microgrid’s frequency by adjusting the power output of the MT, DEG, and FC.

The combination of FOVSG with SMES unit aids to augment the microgrid’s frequency consistency. The uncertainty due to load, solar and wind are considered to imitate realistic operation of microgrid. The distributed generation sources are represented by a transfer function model^3^, which is given by Eq. (1) to (5).

1 $$\:{G}_{MT}\left(s\right)=\:\frac{{K}_{MT}}{(1+s{T}_{MT})}$$

2 $$\:{G}_{DEG}\left(s\right)=\:\frac{{K}_{DEG}}{(1+s{T}_{g})(1+s{T}_{t})}$$

3 $$\:{G}_{FC}\left(s\right)=\:\frac{{K}_{FC}}{(1+s{T}_{FC})(1+s{T}_{inv})(1+s{T}_{IC})}$$

4 $$\:{G}_{PV}\left(s\right)=\:\frac{{K}_{PV}}{(1+s{T}_{PV})}$$

5 $$\:{G}_{WTG}\left(s\right)=\:\frac{{K}_{WTG}}{(1+s{T}_{WTG})}$$

where, K_MT_, K_DEG_, K_FC_, K_PV_, and K_WTG_ are the gains of MT, DEG, FC, PV, and WTG respectively.

T_MT_, T_FC_, T_PV_, and T_WTG_ are the time constant of MT, FC, PV, and WTG respectively.

T_g_ is the governor’s time constant.

T_t_ is the turbine’s time constant.

T_inv_ is the Inverter’s time constant.

T_IC_ is the Interconnection device time constant.

The following Eq. (6) describes the generator and load model’s transfer function^3^.

6 $$\:{G}_{sys}\left(s\right)=\:\frac{{K}_{sys}}{2Hs+D}$$

Where, K_sys_ is the characteristic constant for system frequency.

H is the constant of inertia in the system.

D is the system’s damping coefficient.

## Modelling of FOVSG with SMES unit

A power differential occurs in conventional power systems whenever there is a mismatch between the system’s output power and the power required by the loads. During this time interval, the rotational kinetic energy stowed within the mechanical component of the synchronous generator’s rotor serves to offset this discrepancy, leading to a reduction in the rotational speed of the synchronous generator. The system’s frequency deviates from its originally rated value based on the synchronous generator’s speed. Due to the lack of rotational kinetic energy in distributed energy source inverters, the range of frequency variation will unavoidably rise as their power penetration level improves. To solve the aforementioned issues, distributed energy source inverters can be designed to mimic the rotational inertia of synchronous generators by adjusting their power output based on the system’s frequency changes. This represents the fundamental idea behind VSG technology.

However, the system encounters output power oscillations and frequency fluctuations due to an increase in system order resulting from the incorporation of virtual inertia. This can be overcome with the help of the FOVSG technique, in which fractional virtual inertia is introduced to reduce the system order. As a result, FOVSG can suppress active power oscillations and frequency fluctuations. The active power control law^1^, which is essential for emulating inertial response, is expressed in Eq. (7) as follows:

7 $$\:{P}_{m}-\:{P}_{e}={J\omega}_{0}\frac{d\omega\:}{dt}+D(\omega\:-{\omega}_{0})$$

Where, ω_0_ is the rated angular frequency.

ω is the output angular frequency.

P_m_ is the mechanical power.

P_e_ is the output power.

J is the rotational inertia.

D is the damping coefficient.

The above Eq. (7) indicates the existence of two degrees of freedom, denoted by J and D. As a result, there is a severe limitation on the flexibility needed to offer sufficient active power regulation and inertial response concurrently. These competing goals serve as the impetus for employing the Fractional Order Control (FOC). By implementing FOC, the Eq. (7) can be reformulated^1^ as follows:

8 $$\:{P}_{m}-\:{P}_{e}={J\omega}_{0}\frac{{d}^{\lambda\:+\gamma\:}{\omega}_{f}}{dt}+{D}_{1}\frac{{d}^{\gamma\:}{\omega}_{f}}{dt}+{D}_{2}{\omega}_{f}$$

Where, ω_f_ is the fractional operator-generated frequency.

D_1_ and D_2_ are the virtual rotor constant.

λ and γ are the two fractional numerals.

The following Eq. (9) represents the transfer function^1^ between frequency (Δω) and active power increment (ΔP).

9 $$\:G\left(S\right)=\frac{\varDelta\:\omega\:}{\varDelta\:P}=\:-\:\frac{1}{{J\omega}_{0}{s}^{\lambda\:+\:\gamma\:}+\:{D}_{1}{s}^{\gamma\:}+\:{D}_{2}}$$

Where, λ and γ ranges from 0 to 1, and their summation must be equal to unity.

The virtual rotor model of FOVSG can be formulated with the help of the above Eq. (9) as follows:

10 $$\:{P}_{REF}=({H}_{f}{s}^{\lambda\:+\:\gamma\:}+\:{D}_{1}{s}^{\gamma\:}+\:{D}_{2})\varDelta\:f$$

Where, H_f_, D_1_, and D_2_ are the FOVSG parameters of virtual rotor.

P_REF_ is the inverter’s power reference.

Δf is the change in frequency.

The output power of the virtual control system (ΔP_pi_) is denoted^3^ as follows:

11 $$\:{\varDelta\:P}_{pi}=-\left(\frac{1}{{R}_{f}}+\frac{{K}_{f}}{s}\right)\varDelta\:f$$

Where, R_f_ and K_f_ are the FOVSG parameters of virtual primary and secondary control respectively.

The energy storage in the SMES unit is facilitated by the magnetic coil and constructed using a superconducting material characterised by fewer resistances. The maintenance of the SMES coil in a superconducting state ensures the absence of energy loss, hence leading to a high level of efficacy. The SMES unit comprises a superconducting coil that necessitates cooling through the use of liquid helium. Additionally, a power conversion system (inverter/converter) links the superconducting coil to the grid, where it is charged to a preset value during normal grid operation that is far lower than its maximum charge. In the event of an abrupt increase in demand, the stored energy is returned to the grid using the power conversion system. After satisfying the requisite demands, the coil recharges to its starting current. The SMES technology has been identified as the most appropriate option for augmenting frequency steadiness owing to its numerous advantages, including limitless charging and draining cycles, rapid response duration, high efficacy, and extended lifespan^3^. However, commercial expansion has been impeded by the high expense, which is mostly attributable to cryogenic cooling. SMES coil production and related auxiliary components have become less expensive in recent years due to better manufacturing techniques and the usage of more widely available materials with comparable superconducting qualities. This has made it possible for prices to vary greatly based on the component utilised. Power costs between 130 and 515 $/kW, while energy costs between 700 and 10,000 $/kWh^13^.

**Fig. 2:**
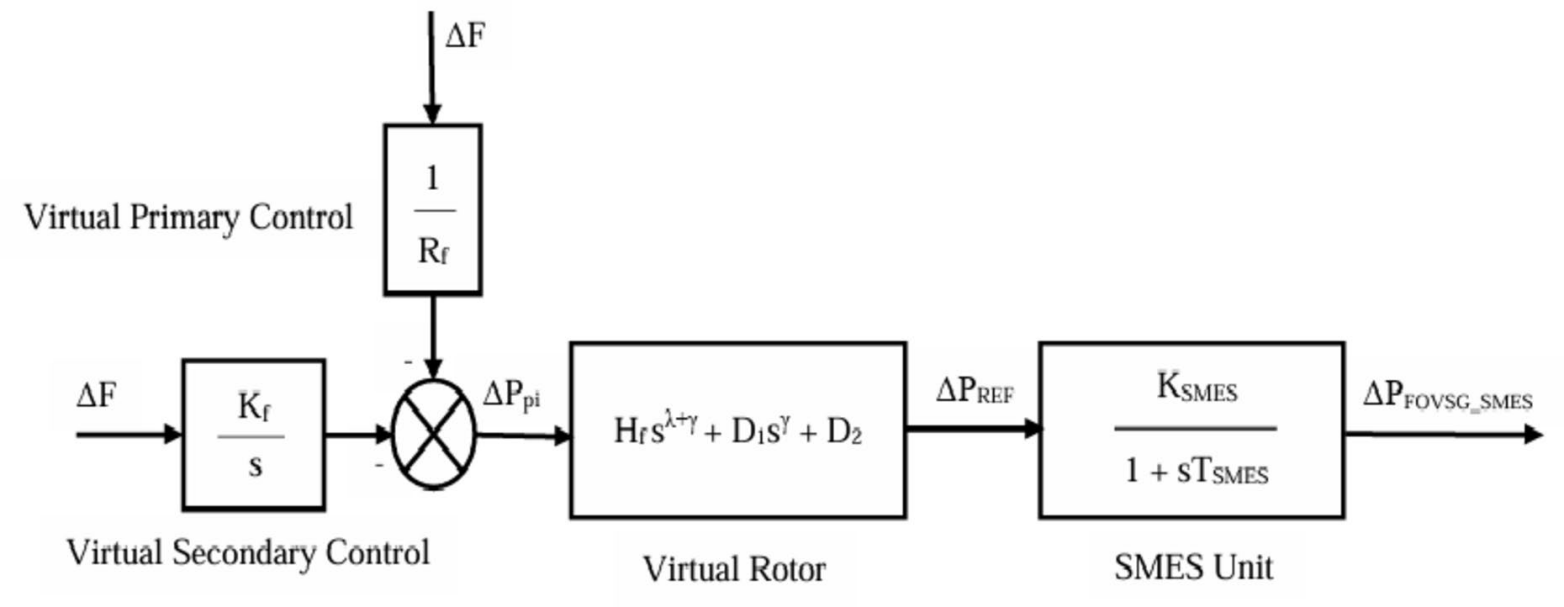
Mathematical model of FOVSG with SMES unit.

The SMES unit can be expressed mathematically^3^ in Eq. (12), and Fig. 2 depicts the mathematical model of FOVSG-based SMES unit.

12 $$\:{G}_{SMES}\left(s\right)=\:\frac{{K}_{SMES}}{(1+s{T}_{SMES})}\:$$

Where, K_SMES_ and T_SMES_ are the gain and time constant of SMES unit respectively.

### African vulture optimization algorithm

The African Vulture Optimization Algorithm^17^ was created by B. Abdollahzadeh et al. in 2021, and it replicates the navigating and foraging behaviours exhibited by African vultures. The reduced computing complexity and enhanced versatility of AVOA make it a powerful and effective metaheuristic algorithm. There are four distinct steps involved in the process of AVOA, which are described as follows:

#### Step – 1: identification of the finest vulture in any group

Once the starting population has been established, the suitability of each solution is evaluated. The most suitability solution is designated as the finest vulture in the first group, while the second-highest suitability solution is designated as the finest vulture in the second group. The remaining solutions in both groups are then adjusted using Eq. (13) in order to move closer to the best solutions in their respective groups. During each iteration of the fitness evaluation process, the entire population undergoes a recalculation.

13 $$R\left(i\right)=\left\{\begin{array}{l}{Best\:Vulture}_{1\:}{if\:p}_{i\:}{=L}_{1\:}\\{Best\:Vulture}_{2\:}{if\:p}_{i\:}{=L}_{2\:}\end{array}\right.$$

Where, R(i) is the i_th_ solution’s finest vultures.

Best Vulture_1_ and Best Vulture_2_ are the first and second group’s finest vultures.

L_1_ and L_2_ are the limits with a sum of one in the interval [0, 1].

The value of pi is derived using the Roulette wheel method, which involves selecting the probabilities of the finest solutions for each group as defined in Eq. (14).

14 $$\:{p}_{i}=\frac{{F}_{i}}{\sum\:_{i=1}^{n}{F}_{i}}\:$$

Where F_i_ is the fitness value of i_th_ vulture.

#### Step – 2: evaluation of vulture’s hunger rate

Vultures often seek food when they are full and energised, as this extends their foraging range. However, when starving, they can’t keep up with the stronger vulture in its search for food and can’t fly very far. Consequently, they get violent when they are hungry. The mathematical representation of this behaviour is depicted in Eq. (15).

15 $$\:F=\left(2*{r}_{1}+1\right)*z*\left(1-\frac{{iteration}_{i}}{maxiterations}\right)+t$$

Where, F is the contentment level of vultures.

r_1_ is the random numeral between 0 and 1.

z is the random numeral between − 1 and 1.

iteration_i_ is the current iteration.

Maxiterations is the maximum iterations.

The value of ‘t’ in the preceding Eq. (15) is determined by the subsequent Eq. (16).

16 $$\:t=h*\left\{{sin}^{w}\left(\frac{\pi\:}{2}*\frac{{iteration}_{i}}{maxiterations}\right)+cos\left(\frac{\pi\:}{2}*\frac{{iteration}_{i}}{maxiterations}\right)-1\right\}$$

Where, h is the random numeral between − 2 and 2.

w is the fixed numeral.

#### Step – 3: exploration

The execution of the exploration and exploitation step is contingent upon the values of the contentment level of vultures (F). If |F| is greater than one, AVOA enters the exploration step. The vultures with superior eyesight and intelligence are able to locate food and identify the impoverished dying animals. Finding food is difficult for vultures. They carefully evaluate their dwelling habitat before moving far for food. Vultures can randomly survey many locations using two approaches. AVOA’s parameter P_1_, which ranges from 0 to 1, is utilised to pick between the two approaches at this step. This phenomenon is characterised by the following Eq. (17).

17 $$\:P(i+1)=\left\{\begin{array}{l}{(R\left(i\right)-\left(\left|X*R\left(i\right)-P\left(i\right)\right|\right)*F)\:if\:P}_{1\:}{\ge\:r}_{P1}\\\:{R\left(i\right)-F+r}_{2\:}{*(\left(ub-lb\right)*r}_{3\:}+lb)\:{if\:P}_{1\:}{<r}_{P1\:}\end{array}\right.$$

Where, P(i + 1) is the vulture’s position in the succeeding iteration.

R(i) is one of the premier vultures.

X is the distance that vultures travel to protect their food from others.

P(i) is the vulture’s current vector position.

ub & lb are the upper and lower limits of the search space.

r_P1_, r_2_ & r_3_ are the random numerals between 0 and 1.

#### Step – 4: exploitation

The AVOA’s final step is the exploitation phase, which comprises two distinct approaches; each of them is selected according to two specific limits, namely P_2_ and P_3_. If |F| is between 0.5 and 1, the AVOA begins the first phase of the exploitation. A random numeral (r_P2_) ranging from 0 to 1, is produced at the commencement of this phase. The Siege-fight approach is used when P_2_ ≥ r_P2_; otherwise, the rotating flight approach is employed. The first stage of exploitation is mathematically expressed in Eq. (18).

18 $$P(i+1)=\left\{\begin{array}{l}{\left(\left|X*R\left(i\right)-P\left(i\right)\right|\right)*\left(F+{r}_{4}\right)-(R\left(i\right)-P\left(i\right))\:if\:P}_{2\:}{\ge\:r}_{P2}\\ R\left(i\right)-R\left(i\right)*\left(\frac{P\left(i\right)}{2\pi\:}\right){\left[{r}_{5\:}{*cos\left(P\left(i\right)\right)+r}_{6\:}*sin\left(P\left(i\right)\right)\right]\:if\:P}_{2\:}{<r}_{P2\:}\end{array}\right.$$

Where, r_P2_, r_4_, r_5_ & r_6_ are the random numerals between 0 and 1.

The siege and violent struggle for food commences in the second phase of the exploitation. This phase begins if |F| is lesser than 0.5. A random numeral (r_P3_) ranges from 0 to 1, is generated at the initial stage of this phase. when P_3_ ≥ r_P3_, the approach entails the accumulation of multiple species of vultures in proximity to the food source. Otherwise, the hostile siege-fight approach is used. The second phase of the exploitation is represented by the following Eq. (19).

19 $$P\left(i+1\right)=\left\{\begin{array}{l}{\frac{1}{2}\left[{BV}_{1}\left(i\right){+BV}_{2}\left(i\right)-\left(\frac{{BV}_{1}\left(i\right)*P\left(i\right)}{{BV}_{1}\left(i\right)-{P\left(i\right)}^{2}}+\frac{{BV}_{2}\left(i\right)*P\left(i\right)}{{BV}_{2}\left(i\right)-{P\left(i\right)}^{2}}\right)*F\right]\:if\:P}_{3\:}{\ge\:r}_{P3\:}\\ R\left(i\right)-\left(\left|R\left(i\right)-P\left(i\right)\right|\right)*F*LF\left(d\right)\:{if\:P}_{3\:}{<r}_{P3}\end{array}\right.$$

Where, r_P3_ is the random numeral between 0 and 1.

BV_1_(i) is the current iteration’s finest vulture from the 1st group.

BV_2_(i) is the current iteration’s finest vulture from the 2nd group.

LF is the function of levy flight.

d is the dimension of the problem.

The term ‘LF’ employed in the preceding Eq. (19) to improve the effectiveness of the AVOA, which is designed as follows.

20 $$\:LF\left(d\right)=0.01*\left(\frac{u*{\left[\frac{\varGamma\:\left(1+\beta\:\right)*sin\left(\frac{\pi\:\beta\:}{2}\right)}{\varGamma\:\left(1+2\beta\:\right)*\beta\:*2\left(\frac{\beta\:-1}{2}\right)}\right]}^{\frac{1}{\beta\:}}}{{\left|v\right|}^{\frac{1}{\beta\:}}}\right)\:$$

Where, u and v are the random numerals between 0 and 1.

β is the constant (1.5).

**Fig. 3:**
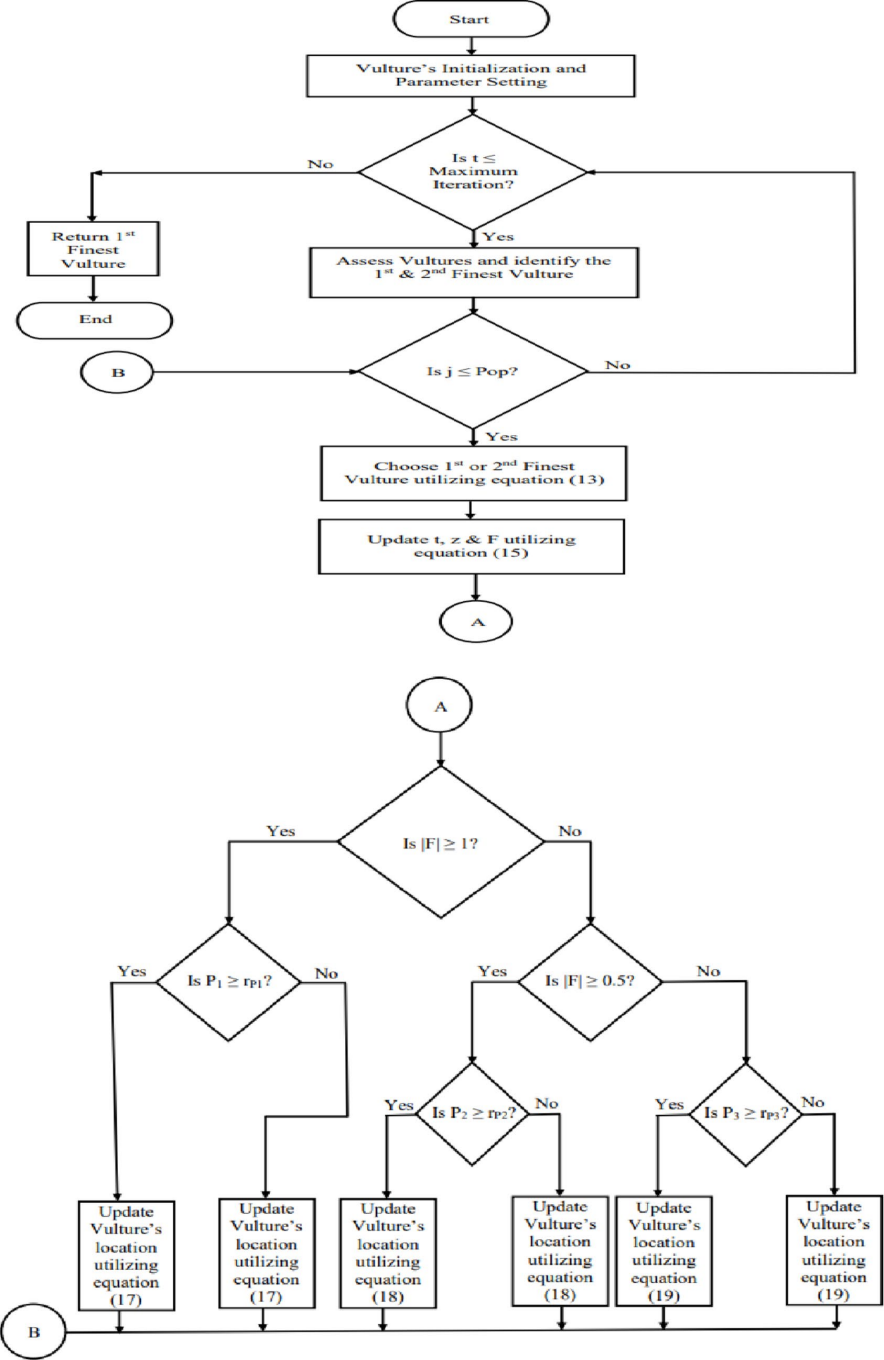
AVOA flowchart.

The preceding Eqs. (13)–(20) are obtained from^17^. The flowchart of AVOA^17^ is shown in Fig. 3 for better understanding.

### FOPID controller

The power network’s performance relies on selecting the controller and objective function. The FOPID controller is highly suitable for the frequency regulation study due to its desirable qualities, including the ability to eliminate steady state error, exhibit robustness in the face of plant gain changes, and effective disturbance rejection^32^. Furthermore, increasing the control parameters results in enhancing the system’s performance to meet the desired level^21^. Hence, the authors have selected the FOPID controller for this study. The proposed FOPID controller model is designed with the help of Oustaloup approximation theory to approximate the fractional order calculus operator and the frequency band chosen as [0.001,1000], with a filter order of 5. The below Eq. (21) provides the mathematical representation of the FOPID controller.

21 $$\:G\left(s\right)= {K}_{P}+\:\frac{{K}_{I}}{{s}^{\lambda}}+{K}_{D}{s}^{\mu\:}$$

Where, K_P_, K_I_ & K_D_ are the FOPID controller’s gain parameters.

λ & µ are the FOPID controller’s fractional parameters.

The ITAE criterion is employed as the objective function in this work since it offers better performance compared to other performance metrics^21^. The ITAE objective function^3^ is expressed as follows:

22 $$\:J={\int}_{0}^{t}t*\left|\varDelta\:f\right|*dt$$

Where, J is the performance Index.

t is the simulation time.

Δf is the frequency variation.

## Simulation outcomes and discussion

**Fig. 4:**
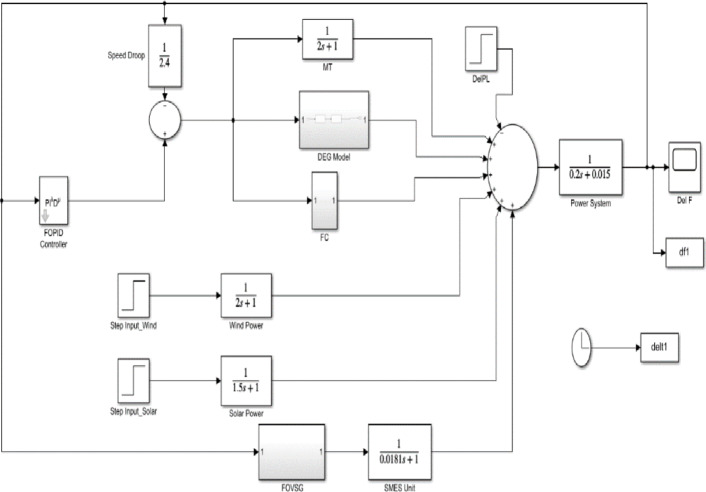
Simulation model of FOVSG with an SMES unit for microgrid frequency regulation.

**Fig. 5:**
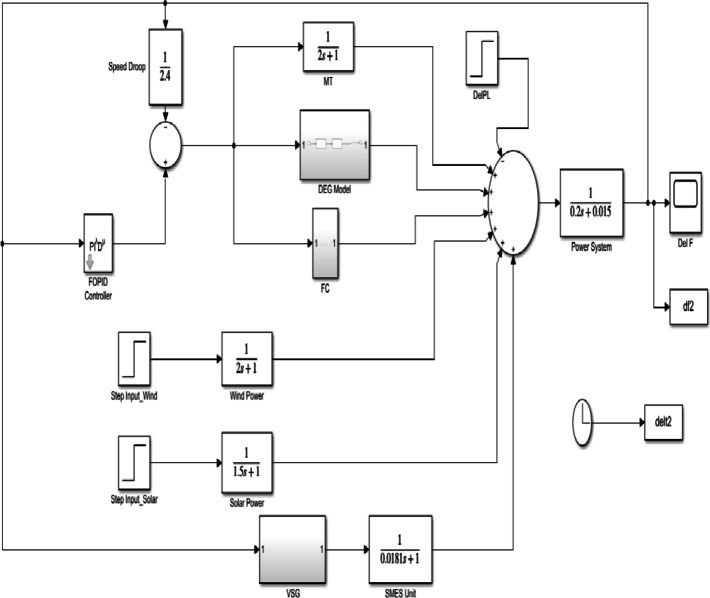
Simulation model of traditional VSG with an SMES unit for microgrid frequency regulation.

The simulation model of FOVSG with an SMES unit for microgrid frequency regulation is represented in Fig. 4, and the simulation model of traditional VSG with an SMES unit for microgrid frequency regulation is represented in Fig. 5. The suggested frequency response models are simulated in MATLAB software, and the system parameters used for the simulations are mentioned in the Appendix. In order to achieve the desired performance, it is essential to appropriately tune the parameters of FOVSG and FOPID controller for effective frequency regulation in response to variations in operating conditions. Thus, a recently developed metaheuristic algorithm called AVOA is utilized in this study to adjust the FOPID controller and FOVSG parameters via the ITAE criterion. The significant parameter choices of the suggested algorithm are indicated in Table 1. In the FOPID controller’s parameter tuning, the gain parameters (K_P_, K_I_, and K_D_) vary from − 10 to 10, whereas fractional parameters (λ & µ) range from 0 to 2. In the case of FOVSG parameter tuning, the virtual secondary control parameter (K_f_) has a limit of 0 to 10, and the virtual rotor constant (D_1_ and D_2_) ranges from 0 to 100, whereas the virtual inertia constant (H_f_) and the virtual primary control parameter (R_f_) are assigned as 0.9 pu s and 5 pu Hz/MW[3]. Based on the hit-and-trial approach, the fractional parameters of FOVSG (λ & γ) are chosen as 0.75 and 0.25, respectively.

**Fig. 6:**
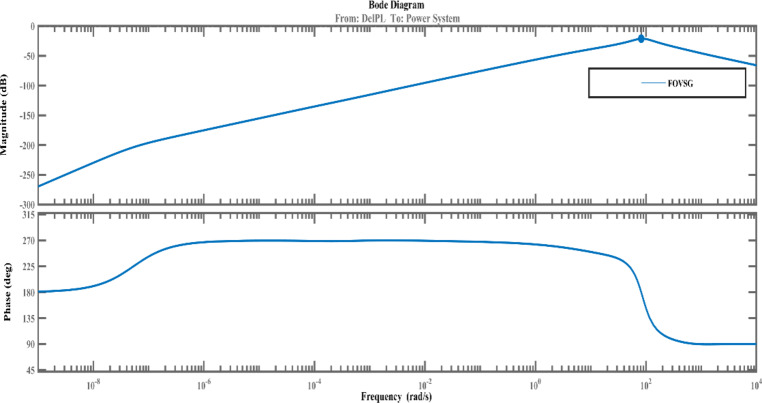
Bode diagram of FOVSG system.

**Fig. 7:**
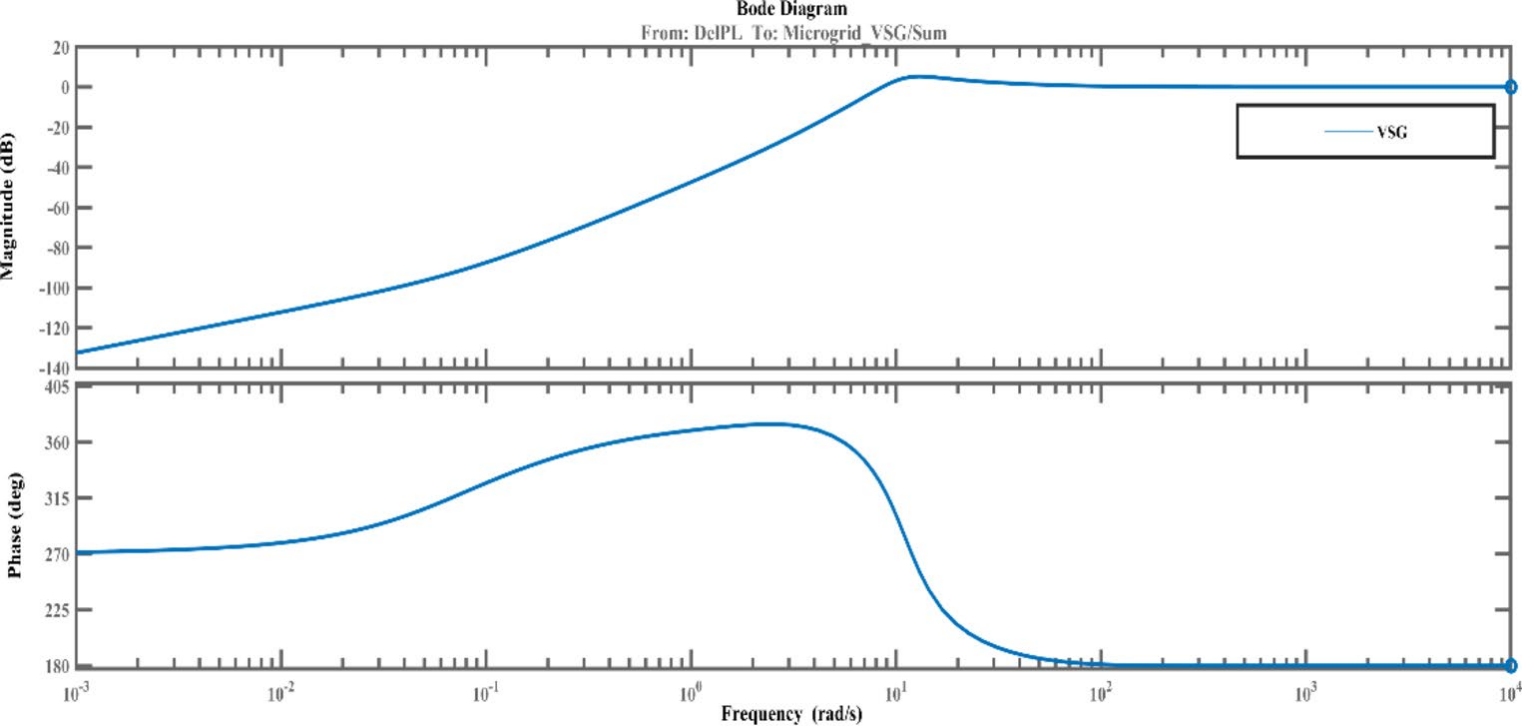
Bode diagram of VSG system.

The suggested FOVSG with SMES unit for microgrid frequency regulation is contrasted against the traditional VSG with SMES unit to demonstrate its superior performance. Consequently, the traditional VSG parameters are tuned by the AVOA using the ITAE criterion. The traditional VSG with SMES unit model for frequency control of microgrids is obtained from^3^. The virtual secondary control parameter (K_i_) varies from 0 to 10, and the virtual rotor constant (D_i_) varies from 0 to 100, whereas the virtual inertia constant (H_i_) and virtual primary control parameter (R_i_) of traditional VSG parameters are assigned as 0.9 pu s and 5 pu Hz/MW^3^. The effectiveness of the proposed system is tested under multiple scenarios by considering the variation in renewable sources (PV & wind) and load. The aforementioned maximum and minimum ranges for the various parameters of FOPID and FOVSG are determined by simulation-based approach and from the literatures^1,3,8] and [32^. Additionally, the suggested FOPID controller’s performance is verified by comparing it to a traditional PID controller in the same circumstances. In addition, the stability of the proposed frequency regulation model in an isolated microgrid (both FOVSG and VSG systems for the combination of SMES and FOPID controller) is examined using a bode plot in MATLAB / Simulink. The relevant bode diagram are shown in Figs. 6 and 7 and it revealed that the suggested systems (FOVSG and SMES combination with FOPID controller) have a higher gain margin (21.2dB at frequency 81.9 rad/s) than the other traditional VSG systems, indicating that the proposed system is more stable.

**Table 1:** Significant parameters setting.

Algorithm Name	Parameters Choices
AVOA	Size of population as 20 Maximum number of iterations as 100 L_1_ as 0.8 L_2_ as 0.2 w as 2.5 P_1_ as 0.6 P_2_ as 0.4 P_3_ as 0.6

### Scenario-I

**Fig. 8:**
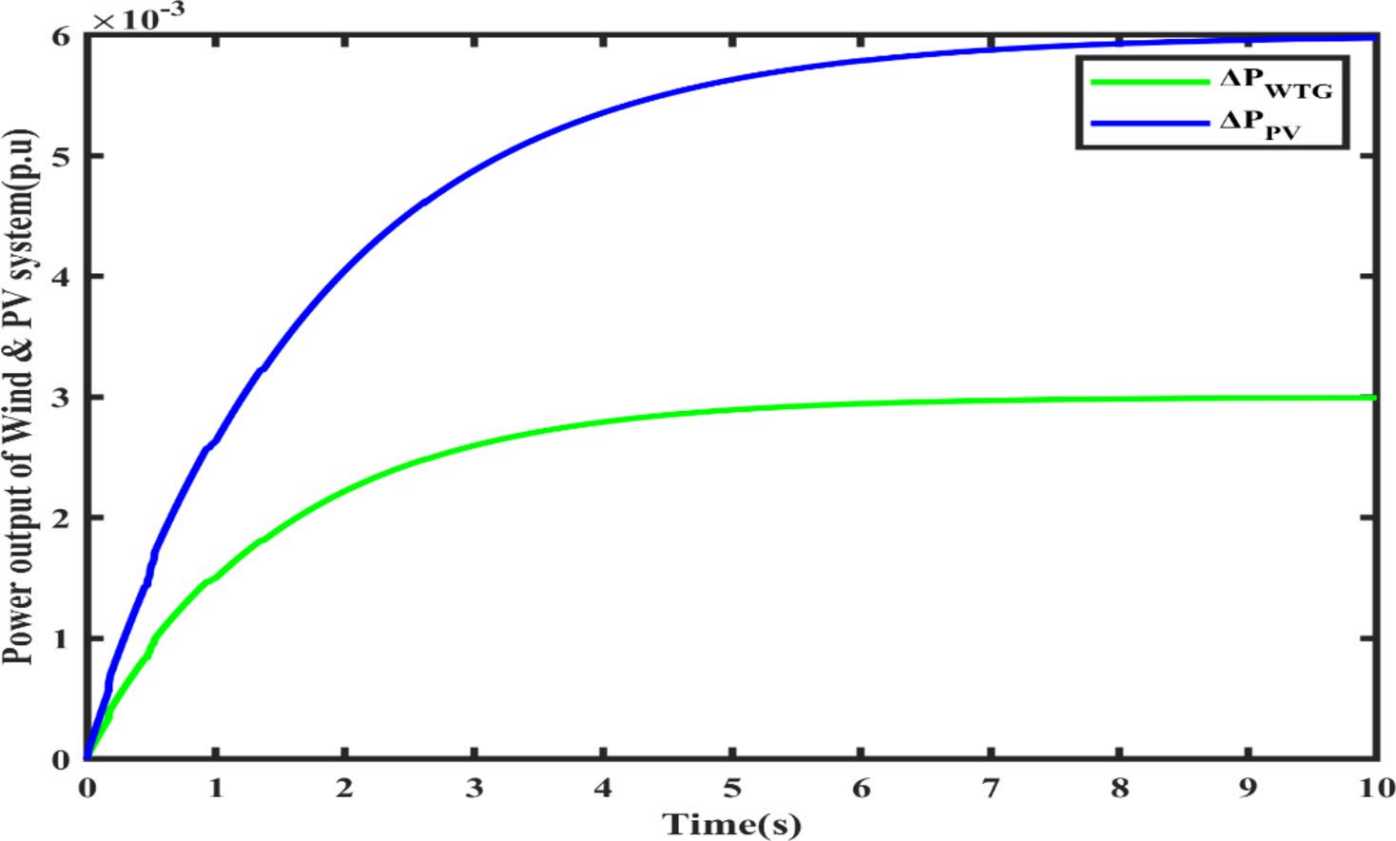
Power output of Wind and PV systems.

**Fig. 9:**
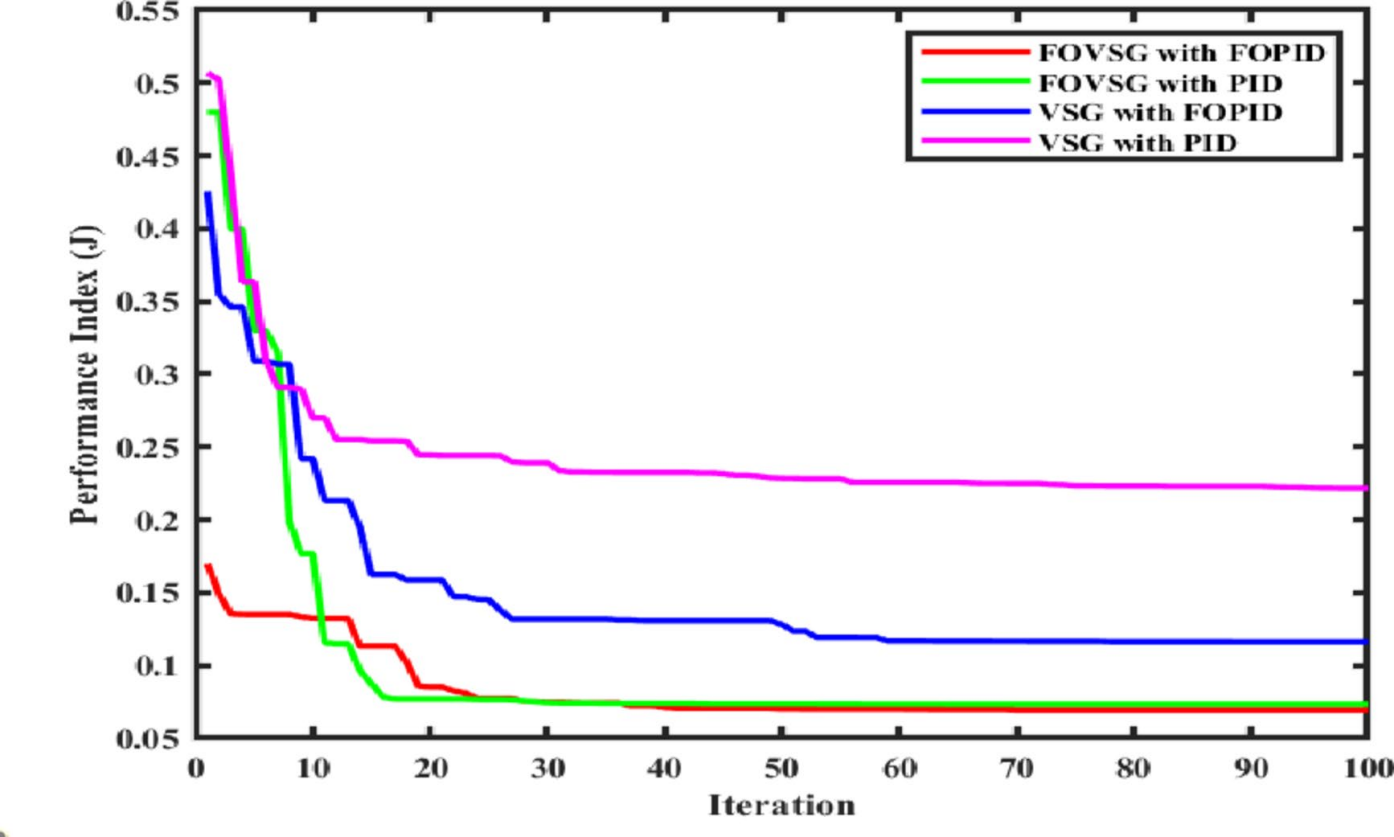
Convergence curve.

**Fig. 10:**
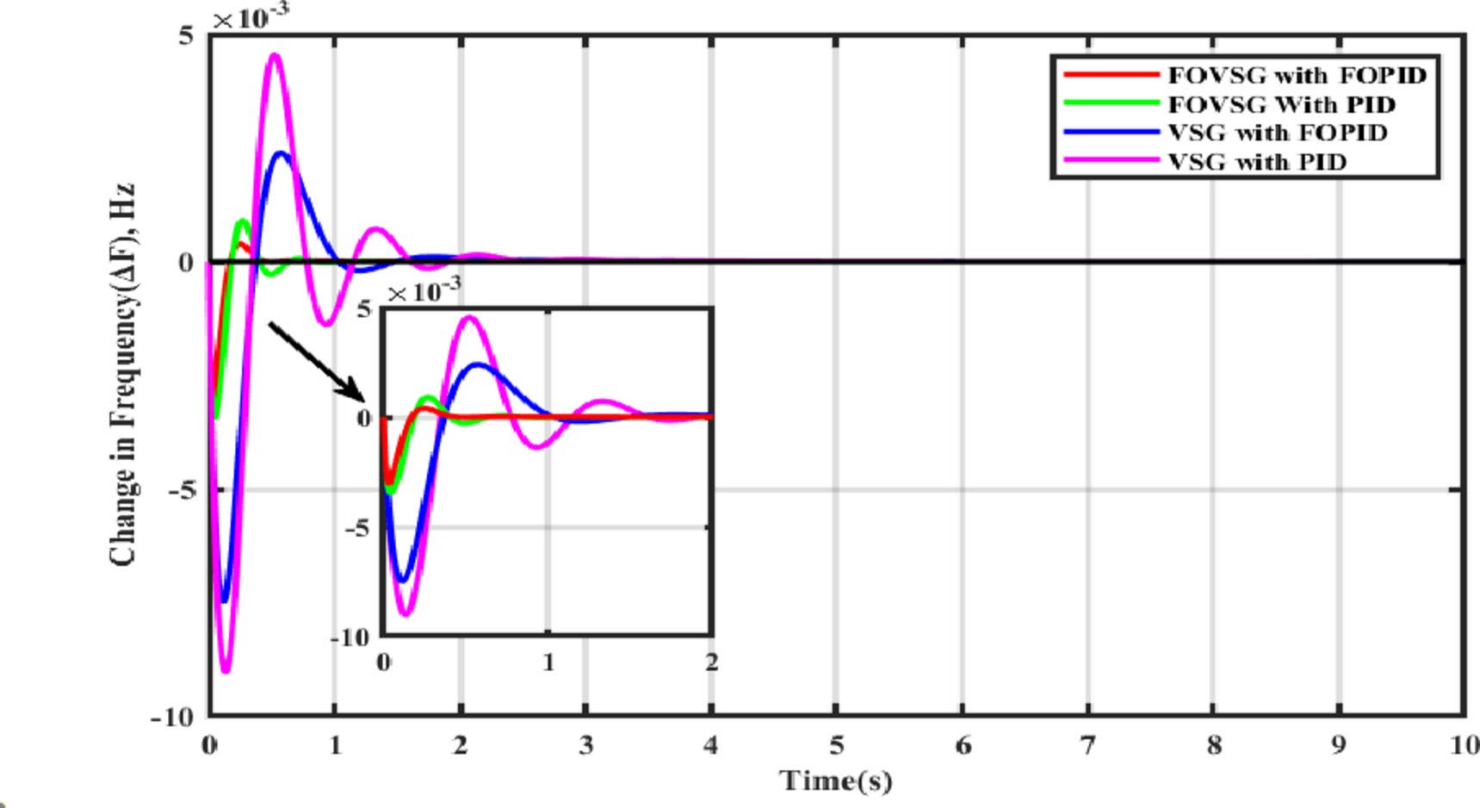
Frequency response for Scenario-I (Presence of step load disturbances, PV & wind power output).

In this scenario, both systems (FOVSG with SMES unit and traditional VSG with SMES unit for microgrid frequency regulation) are simulated under a step load perturbation of 0.05 p.u. with ΔP_PV_ and ΔP_WTG_ kept constant, and the corresponding power output of the Wind and PV systems is depicted in Fig. 8. The suggested algorithm (AVOA) is utilized to optimize the FOPID and PID controller’s parameters, FOVSG parameters, and traditional VSG parameters. The 30 individual runs are made for each system, and the best outcomes are recorded. The optimized parameters for the various systems are tabulated in Table 2, and the associated convergence curves are illustrated in Fig. 9. The time domain frequency responsiveness for the various systems is represented in Fig. 10, and it is evidenced that the FOVSG and SMES unit combination system with FOPID controller for microgrid frequency regulation outperforms the other system related to maximum overshoot, settling period, and frequency fluctuations.

**Table 2:** Optimized parameters for both the systems with different controller.

System Type	Controller Parameters					FOVSG Parameters			Traditional VSG Parameters		Performance Index (J)
	K_*P*_	K_I_	K_D_	λ	µ	K_f_	D_1_	D_2_	K_i_	D_i_	
FOVSG and SMES unit combination with FOPID controller for microgrid frequency regulation	9.4777	9.6800	2.0103	1.1611	0.9679	3.5317	17.5156	48.2567	–	–	0.0694
FOVSG and SMES unit combination with PID controller for microgrid frequency regulation	2.9877	2.1606	1.7318	–	–	2.9374	12.4032	41.7838	–	–	0.0734
Traditional VSG and SMES unit combination with FOPID controller for microgrid frequency regulation	1.3910	9.9856	0.4253	1.0232	0.2836	–	–	–	2.6196	10.5303	0.1163
Traditional VSG and SMES unit combination with PID controller for microgrid frequency regulation	0.7069	9.9483	0.1370	–	–	–	–	–	2.1851	9.9850	0.2217

### Scenario-II

**Fig. 11:**
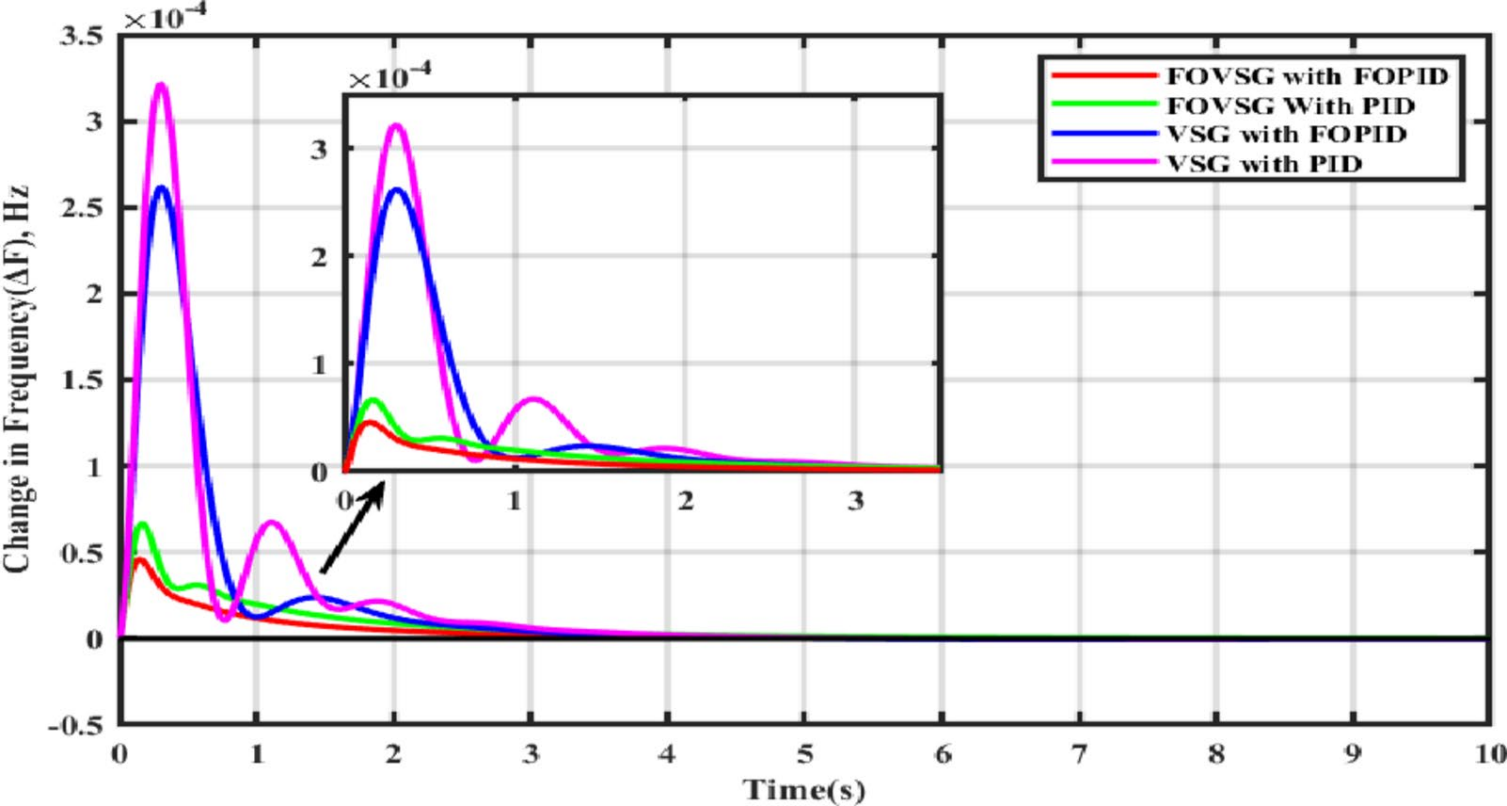
Frequency response for Scenario-II (Presence of PV and wind power output only).

In this scenario, the frequency control models are examined in the presence of changes in RES power output alone (PV as 0.006 p.u. and wind as 0.003 p.u.), whereas on the other hand, there is no load disturbance. The related frequency response characteristics are depicted in Fig. 11, and it is confirmed that the microgrid frequency regulation system consists of an FOVSG and SMES unit combination with FOPID controller, provides greater dynamic response, such as fewer frequency oscillations, lower amplitude, and a shorter settling duration.

### Scenario-III

**Fig. 12:**
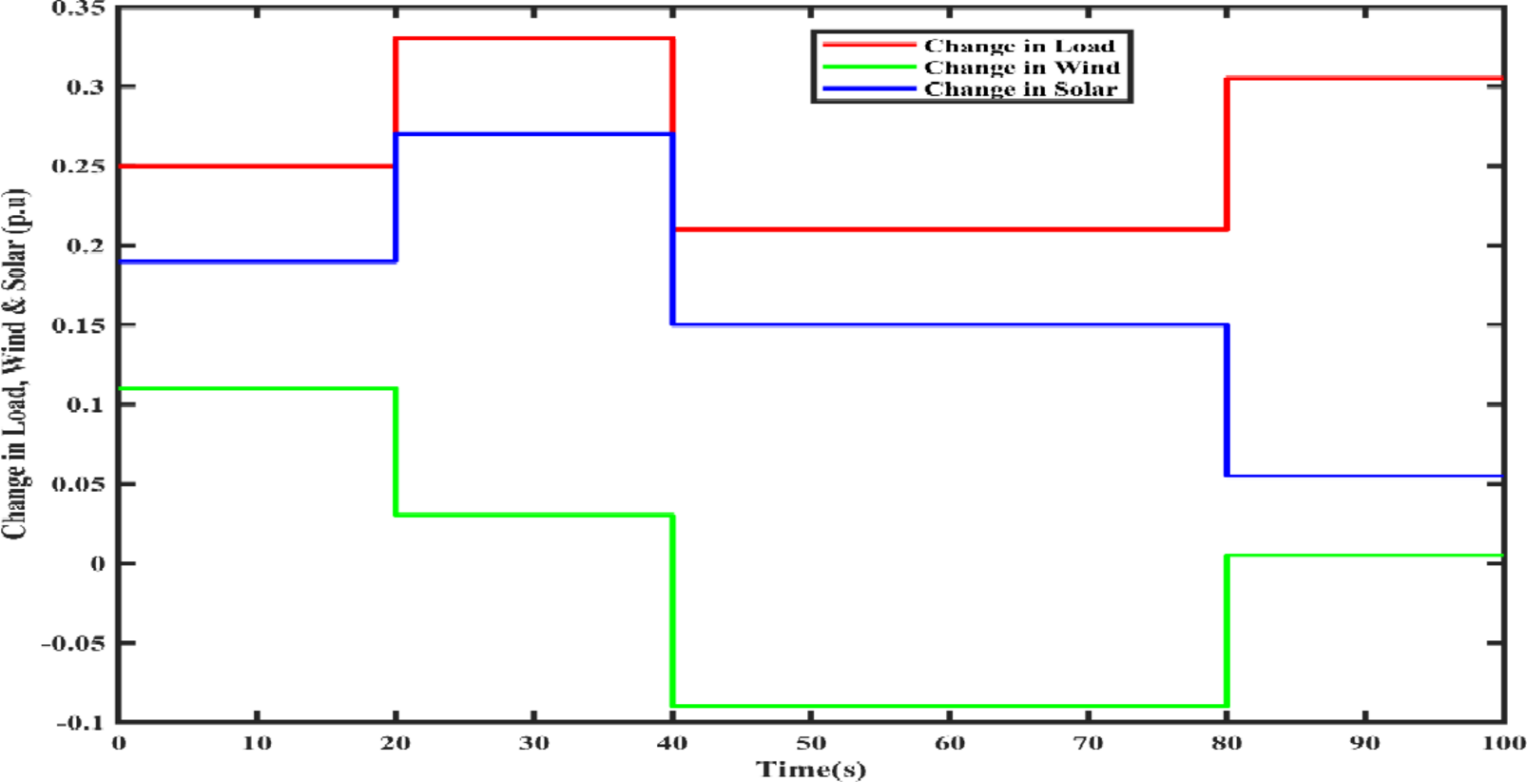
Multi-step disturbances.

**Fig. 13:**
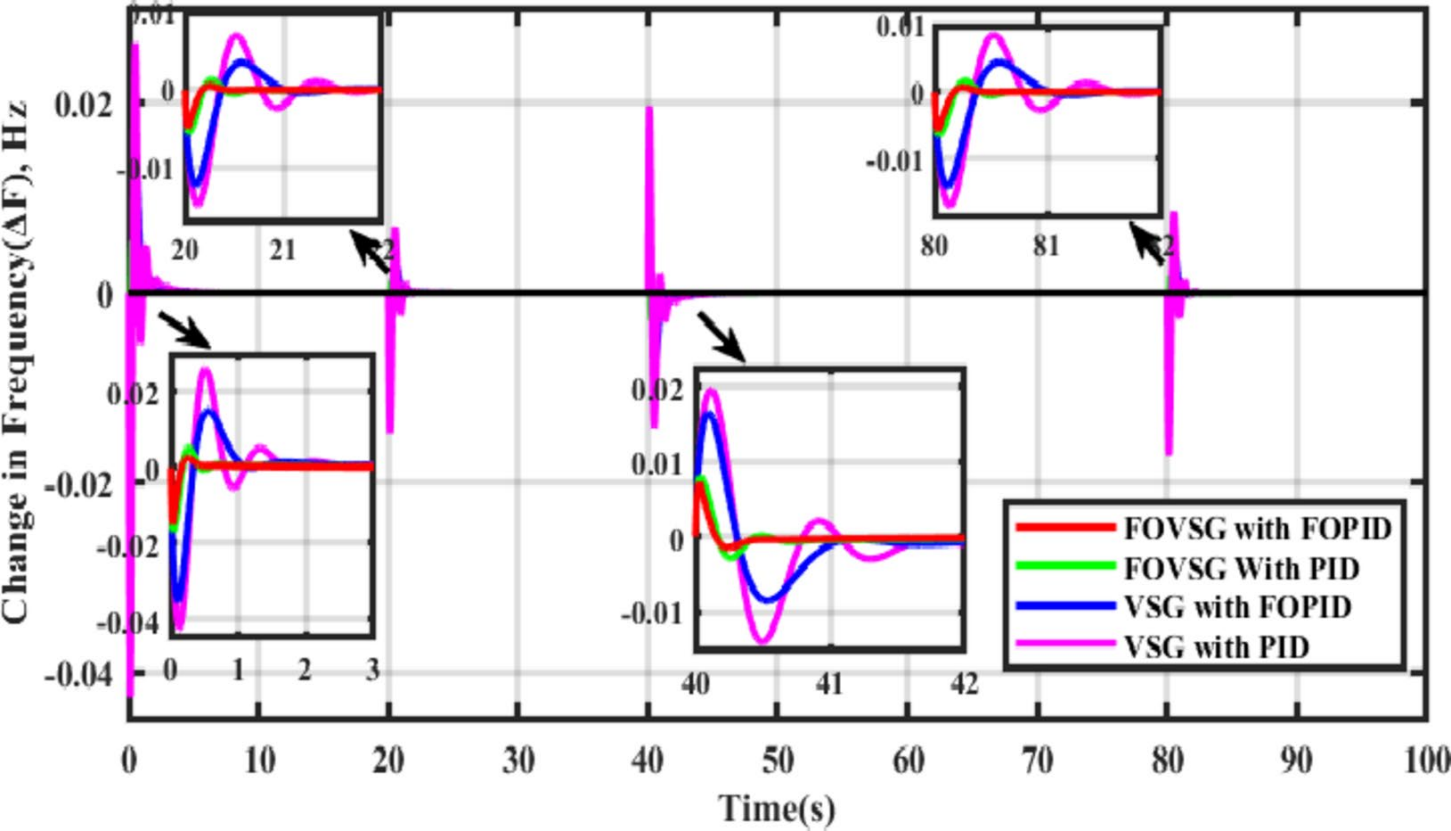
Frequency response for Scenario-III (Presence of Multi-step disturbances).

In order to replicate the variations in uncertainties due to solar irradiation, wind speed, and load changes, multi-step disturbances are considered, which are represented in Fig. 12. The corresponding frequency variations are shown in Fig. 13, and it is illustrated that the suggested microgrid frequency control system using the FOVSG and SMES unit combination with the FOPID controller could effectively eliminate the system frequency changes in the 1st stage beforehand starting the 2nd stage of perturbation at a faster rate, and so on.

### Scenario-IV

**Fig. 14:**
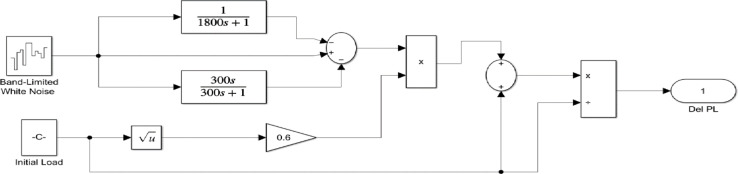
Random Load Model.

**Fig. 15:**
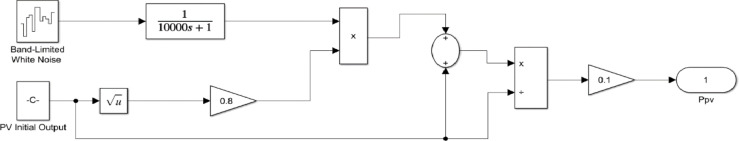
Random PV Model.

**Fig. 16:**
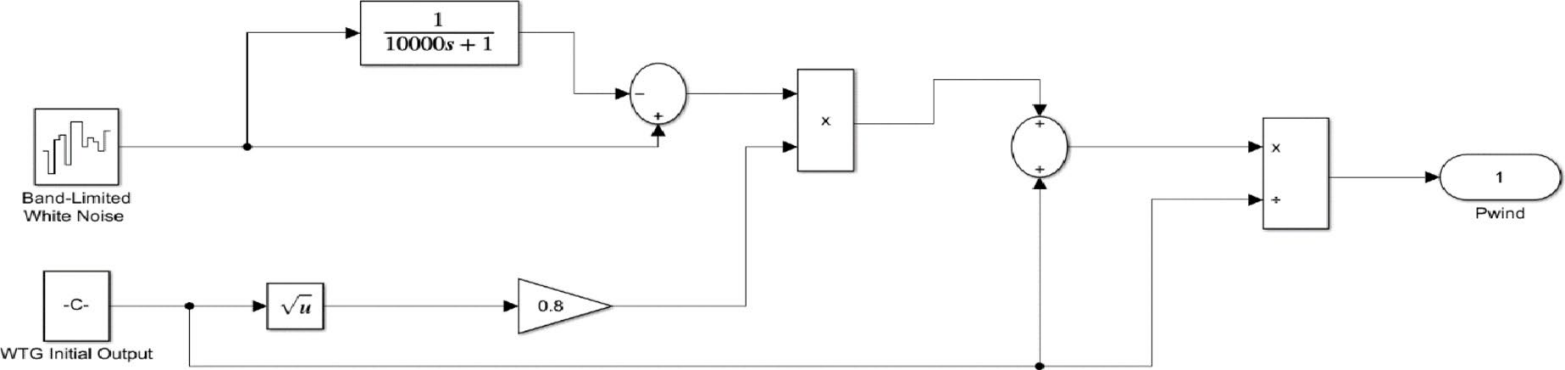
Random wind model.

The severe changes in load, PV and wind power output can be simulated with the help of a random load, PV, and wind model, which are represented^3^ in Figs. 14 and 15, and Fig. 16 respectively. The initial load, Initial power output of PV and wind are considered as 0.1 p.u. The PV and wind power outputs^3^ are determined by the subsequent Eqs. (23) and (24), respectively.

23 $$\:{P}_{PV}=\:\eta\:\varPhi\:S[1-0.005\left(T+25\right)]$$

Where, η is the conversion efficiency (18%) of PV array.

Ф is the solar irradiation (1000 W/m^2^).

S is the PV array’s measured area (1.6 m^2^ for 30-kW system).

T is the ambient temperature (25˚C).

24 $$\:{P}_{WT}=\:\frac{1}{2}\rho\:A{C}_{P}{V}_{W}^{3}$$

Where, ρ is the density of air (1.225 kg/m^3^).

A is the Swept area (314.16 m^2^ for a 100-kW wind turbine).

C_P_ is the co-efficient of power. (0.4)

V_W_ is the Wind velocity (12 m/s).

**Fig. 17:**
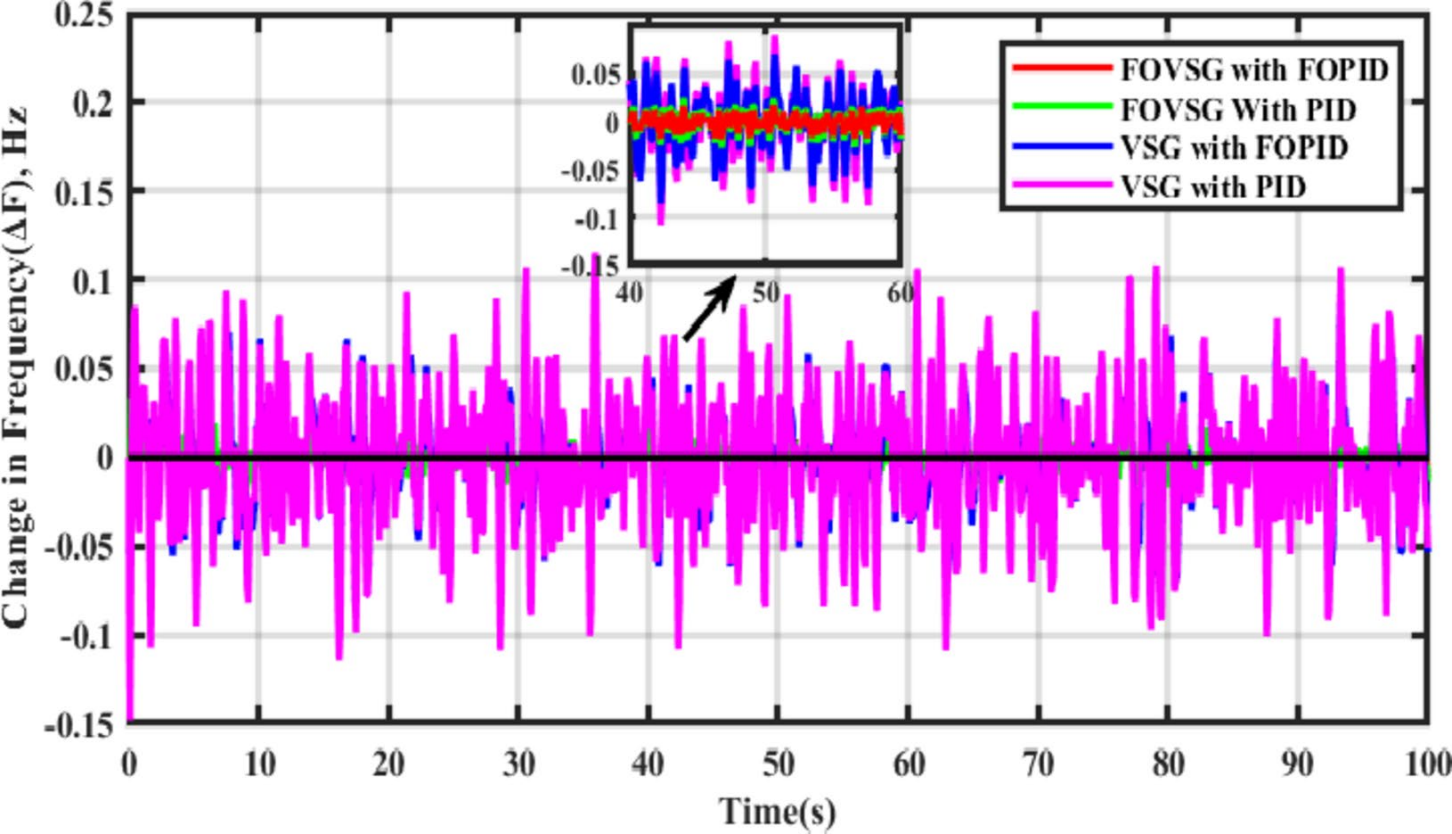
Frequency response for Scenario-IV (Presence of random disturbances).

The time domain frequency fluctuations of the systems are represented in Fig. 17, and it is evident that the proposed system employing the FOVSG and SMES unit combination with the FOPID controller has significant improvements in system dynamics. The numerical analysis of both systems in terms of maximum overshoot and settling duration is described in Table 3.

**Table 3:** Numerical analysis.

Scenarios	Controllers	System using FOVSG with the SMES unit		System using traditional VSG with the SMES unit	
		Maximum Overshoot (Hz)	Settling Duration (s)	Maximum Overshoot (Hz)	Settling Duration (s)
Scenario-I (Consideration of PV, Wind and Load)	FOPID	0.00039	0.41	0.00238	1.54
	PID	0.00089	0.72	0.00454	1.78
Scenario-II (Consideration of PV and Wind only)	FOPID	0.000046	1.86	0.00026	2.59
	PID	0.000065	2.15	0.00032	2.64
Scenario-III (Consideration of Multistep Disturbances)	FOPID	0.00259	0.51	0.01510	1.88
	PID	0.00481	1.34	0.02629	1.91
System with VSG and SMES unit under low inertia condition [20]	PID	–	–	0.005	2.5
System with VSG based SMES unit (Consideration of PV, Wind and Load) [3]	PI	–	–	0.00534	1.89

## Conclusion

In this study, the FOVSG and SMES unit combination with the FOPID controller is utilized in the microgrid frequency regulation model to mitigate the frequency oscillations. The FOPID and PID controllers, FOVSG, and traditional VSG parameters are optimized by a recently developed African vulture optimization algorithm with the aid of the ITAE criterion. The different scenarios are examined to validate the efficacy of the suggested system with different controllers, such as classical PID and FOPID, and the outcomes are contrasted with the other frequency regulation system which consists of a traditional VSG with an SMES unit. The obtained simulation outcomes demonstrated that the frequency regulation model includes the FOVSG and SMES unit combination with the FOPID controller produces superior dynamic performance such as fewer frequency oscillations, reduced peak overshoot, and shorter settling duration. However, our suggested study is restricted to a single-area isolated microgrid. Further, it can be expanded to multi-microgrids with progressive controllers, such as cascaded controllers.

## Electronic supplementary material

Below is the link to the electronic supplementary material.

Supplementary Material 1

## Data Availability

All the data are provided in the manuscript.
