# Supplementary material for: An optimized fractional order virtual synchronous generator with superconducting magnetic energy storage unit for microgrid frequency regulation enhancement
Source: Sci Rep. 2025 Feb 20;15:6209. doi: 10.1038/s41598-025-90483-5 (PMC11842848; doi:10.1038/s41598-025-90483-5)
Supplement: Supplementary file 1 — Supplementary Material 1 [file 41598_2025_90483_MOESM1_ESM.docx]

**Appendix**

| Parameter | Value | Parameter | Value |
| --- | --- | --- | --- |
| K_MT_ | 1 | K_PV_ | 1 |
| T_MT_ | 2 s | T_PV_ | 1.5 s |
| K_DEG_ | 1 | K_WTG_ | 1 |
| T_g_ | 0.4 s | T_WTG_ | 2 s |
| T_t_ | 0.008 s | R | 2.4 pu Hz/MW |
| K_FC_ | 1 | 2H | 0.2 |
| T_FC_ | 0.26 s | D | 0.015 |
| T_inv_ | 0.04 s | K_SMES_ | 1 |
| T_IC_ | 0.004 s | T_SMES_ | 0.0181 s |

**Table A.** System Parameters^3^.
